# Cellular Therapy in Inflammatory Bowel Diseases

**DOI:** 10.3390/jpm16100517

**Published:** 2026-10-03

**Authors:** Samuel Majna, Biljana Brezina, Mohmmed Tauseef Sharip, Sreedhar Subramanian, Nurulamin M. Noor

**Affiliations:** 1 Department of Gastroenterology, Bezrucova Clinic, 811 09 Bratislava, Slovakia; 2 Department of Internal Medicine II—Gastroenterology and Geriatrics, University Hospital Olomouc and Faculty of Medicine, Palacký University Olomouc, 779 00 Olomuc, Czech Republic; 3 Department of Gastroenterology, Cambridge University Hospitals NHS Foundation Trust, Cambridge CB2 0QQ, UK; 4 Department of Medicine, School of Clinical Medicine, University of Cambridge, Cambridge CB2 0AW, UK

**Keywords:** inflammatory bowel disease, cellular therapy, stem cell transplantation, haemopoietic stem cell, mesenchymal stem cell, CAR-T cells, regulatory T cells

## Abstract

Despite significant advances in understanding the biology of inflammatory bowel diseases (IBD), the underlying etiology is still unknown, and currently available treatment options are not curative. Furthermore, even after the advent of multiple targeted “advanced” therapies, approximately one-third of patients do not respond to treatment, and approximately half of patients lose response over time. As a result, there have been efforts made to seek alternative treatment options. In recent years, there have been major advances made in the field of cellular therapy, involving administration of living cells to patients. Notably, the area with the most evidence base to date has been stem cell therapy, with data generated for both mesenchymal and hematopoietic stem cells. More recently, there have also been emerging data exploring the use of chimeric antigen receptor T cells and regulatory T cells. As the landscape for cellular therapy expands in the coming years, an important next step will be to understand which patients may or may not benefit from these therapies and at what point these treatment options might be considered in the disease course of IBD.

## 1. Introduction

Inflammatory bowel diseases (IBD) are increasingly recognized as a large number of diverse phenotypes resulting in chronic, immune-mediated inflammation and damage to the gastrointestinal tract, often resulting in both systemic and extra-intestinal involvement. For both main types of IBD, Crohn’s disease (CD) and ulcerative colitis (UC), there are typically alternating periods of flare and remission, with progression over time, which can have a major impact on quality of life for patients [1,2].

In the absence of a cure, management has typically focused on suppressing inflammatory activity with the goal of maintaining remission. The first drugs used to treat IBD were aminosalicylates, which came into use in the 1930s, followed by corticosteroids in the 1950s and immunomodulators in the 1960s [3]. The discovery of anti-tumor necrosis factor (anti-TNF) infliximab, the first monoclonal antibody to treat IBD in the late 1990s, was considered a major breakthrough [4]. Nowadays, there is a wide range of “advanced” therapies comprising monoclonal antibodies and targeted oral small molecules to treat IBD [5], with a large pipeline of further therapeutics expected to arrive in the coming 10 years.

As treatment options have expanded and treatment strategies have modernized [6,7,8], therapeutic goals have also shifted. A treat-to-target strategy has become widely adopted [9], with increasingly lofty ambitions and recommendations for treatment aims [10]. Nowadays, therapeutic goals are focused on normalizing quality of life for patients and preventing long-term complications caused by IBD [11]. In the future, more aspirational targets may be routinely aimed for, such as histological healing in UC and transmural healing in CD [12,13,14]. It is also possible that in the future, treatment of IBD will be focused on even deeper and higher levels of healing, such as with molecular targets [15].

Despite undeniable progress in the treatment of IBD, it must be acknowledged that even with modern therapeutics, a large proportion of patients are unable to achieve satisfactory disease control [16,17]. In recent years, multiple strategies have been tried to overcome the so-called “therapeutic ceiling” of treatment efficacy and effectiveness [18]. In an effort to better control disease activity, many patients have been initiated on combination immunosuppressive therapy; however, even the use of two or more immunosuppressive drugs has not guaranteed treatment success [19]. Seeking to simultaneously influence multiple inflammatory pathways and achieve durable resolution of inflammation, the concept of cellular therapy for IBD has also emerged.

Cellular therapy (also cell therapy or cell-based therapy) refers to administration of living cells to patients in order to treat a disease. To date, perhaps the largest number of clinical trials utilizing cellular therapy in the treatment of IBD have focused on the use of stem cells. Several stem cell-based therapies have advanced through phase I to phase III clinical trials; however, their results have been variable. More recent additional cellular therapies under investigation in IBD include chimeric antigen receptor (CAR)-T cells and regulatory T cells. There are several other potential cellular therapies at much earlier stages of development and evaluation, including, for example, tolerogenic dendritic cells. However, in this review, we did not consider cellular therapies that have not advanced beyond phase I clinical evaluation [20]. In this article, we discuss the current state of knowledge regarding the use of cellular therapy in the treatment of IBD and attempt to outline the future horizons and challenges that lie ahead.

## 2. Stem Cell Therapy

**Figure 1:**
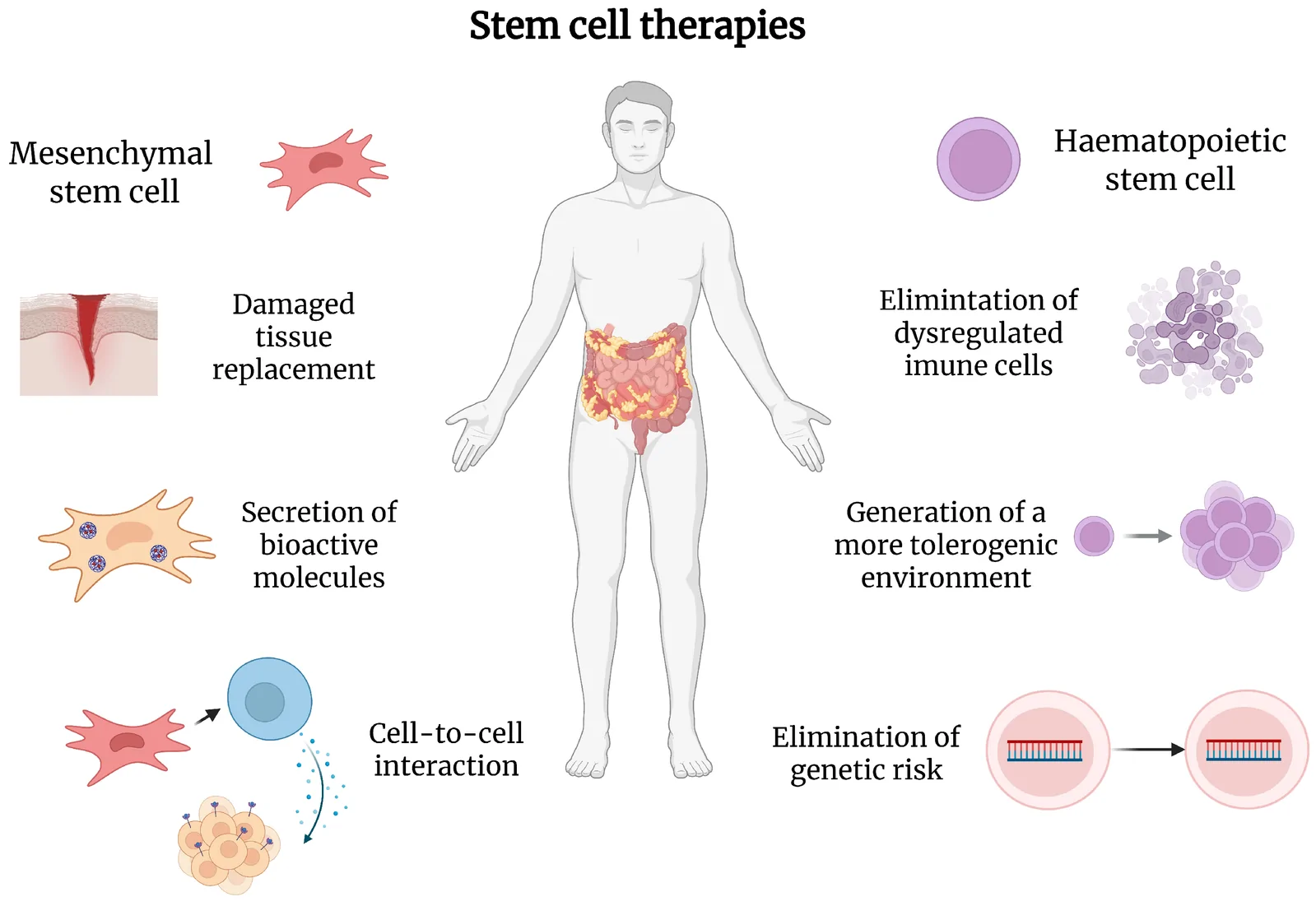
Stem cell therapies in IBD.

Stem cells are generally defined as undifferentiated cells that are capable of self-renewal and can differentiate into specialized cell types, and are involved in the repair and maintenance of tissues. Stem cells are typically classified according to their origin into embryonic, somatic (adult) stem cells, and induced pluripotent stem cells [21]. Research on human embryonic stem cells has been mired in controversy due to legal and ethical issues. Similarly, induced pluripotent stem cells have been associated with oncogenic potential, and this has limited in vivo human research in IBD. As a result, the majority of focus on stem cells in IBD has centered around the use of somatic stem cells as a potential treatment (Figure 1).

Autologous stem cells, which are isolated from the individual for whom they are subsequently intended, are quite distinct from allogeneic stem cells, which are isolated from a genetically different donor—one which is ideally HLA-matched. Moreover, syngeneic cells, although considered allogeneic in some regards because they are received from an identical monozygotic sibling, have often been classified similarly to autologous stem cells [21]. In IBD, mesenchymal stem cells (MSC) and hematopoietic stem cells (HSC) have been tested in the contexts of perianal fistulizing Crohn’s disease (PFCD), refractory luminal IBD, and in the setting of very-early onset IBD (VEO-IBD).

### 2.1. Mesenchymal Stem Cell Therapy

MSCs, also called multipotent mesenchymal stromal cells, have the ability to differentiate into osteoblasts, adipocytes, and chondroblasts and express specific types of CD surface antigens [22,23]. MSCs have been proposed as a therapeutic agent to differentiate and replace damaged tissue, to restore the immune balance and mitigate chronic inflammation either by secreting bioactive molecules or by cell-to-cell interactions. MSCs were initially discovered in the bone marrow, but they can also be found in adipose tissue, umbilical cord, dental pulp, skin, tendon, skeleton, muscle, placenta, synovial, and amniotic fluids [24]. Of these sources, bone marrow and the stromal vascular portion of adipose tissue have been the most extensively studied [25]. MSCs of different sources differ not only in their accessibility, but also in their immunomodulatory capacities, cytokine secretion profiles, and other properties [25,26].

One of the main advantages of MSCs is their low immunogenicity, which has been attributed to their low levels of human leukocyte antigen (HLA) class I expression and the absence of HLA class II expression. Additional factors contributing to low immunogenicity include: lack of co-stimulatory antigens on the surface, e.g., CD80 or CD86 [27], and the ability of MSCs to release substances that suppress cytotoxic T cells, inhibit the activity of dendritic cells [28], and activate regulatory T cells [29]. However, there are several important limitations of using MSCs as a treatment, including their short survival time and poor engraftment after in vivo transplantation [30]. In addition, there is substantial heterogeneity depending on the source of MSCs, preparation methods, doses, routes of administration, and differences in treatment schedules used, all of which may influence therapeutic outcomes. Moreover, there still remains uncertainty about the exact biological mechanisms of action for MSCs, and that, in addition to differentiation and replacement of damaged tissue, MSCs may also act via paracrine signaling to induce secondary immune tolerance and/or impact apoptotic signaling pathways [31].

As well as the MSCs themselves, there has been growing interest in the secretome—comprising both a soluble fraction (cytokines, proteins, growth factors, etc.) and a vesicular fraction (exosomes, microvesicles, and apoptotic bodies). Notably, MSC-derived extracellular vesicles (MSC-EVs) may in the future represent a safer and more effective way to modify the disease than administering MSCs alone [32,33,34,35,36]. However, MSC-EVs, and other similar molecules, are typically considered cell-free therapies and are therefore beyond the scope of this article focusing on cellular therapy.

Two different routes of administration of MSCs have been evaluated in IBD: systemic intravenous administration in the treatment of UC and luminal CD, and local injection of MSCs in patients with PFCD. Firstly, in luminal IBD, there is growing evidence that intestinal inflammation could be decreased through systemic administration of MSCs in animal models [37,38,39,40]. However, the data in luminal IBD to date have been highly variable. Only small pilot studies have been reported using variations in bone marrow-derived and umbilical cord-derived MSCs. The small size of studies, as well as the short-term and subjective nature of outcomes assessed, have precluded any robust conclusions from being able to be drawn, even though there are still ongoing efforts with potentially promising results [41,42,43].

Notably, MSCs have also shown promise in the treatment of the CD-like phenotype of the pouch in patients with UC who underwent restorative proctocolectomy with ileal pouch-anal anastomosis (IPAA) creation [44]. Given the signal in CD-like disease, there has been a resurgence of interest in the role of MSCs in CD. In luminal CD, a phase I trial was performed using autologous bone marrow-derived MSCs for the treatment of patients who were refractory to multiple medical treatments. Only three out of ten patients had a reduction in Crohn’s disease activity index (CDAI) score of at least 70 points; none achieved clinical remission, and seven of the patients experienced several adverse events [45]. These results tempered enthusiasm for MSCs at the time; however, one subsequent phase II trial in patients with refractory luminal CD has suggested possible benefit from administering weekly intravenous infusions of allogeneic MSCs to 16 patients for a period of 4 weeks. Among the 15 patients who completed the study, 12 patients had a clinical response at day 42 (mean reduction in CDAI was 211), 8 patients were in clinical remission, and only one patient had a serious adverse event, which was felt unrelated to the administration of MSCs [46].

Administration of MSCs has reached later phases of clinical trials in the treatment of PFCD. There was much hope and excitement for injection of darvadstrocel (alofisel) MSCs as a biological augmentation strategy for this difficult-to-treat phenotype of CD. Darvadstrocel is a dispersion of expanded adult allogeneic adipose-derived MSCs that showed initially very promising results in ADMIRE-CD. This was a phase III, randomized, double-blinded, placebo-controlled trial, which enrolled 212 patients randomized in a 1:1 ratio to darvadstrocel or placebo. At week 24, 50% of patients treated with darvadstrocel achieved remission, compared with 34% in the placebo group (*p* = 0.024), with similar adverse events in both groups [47]. In extension studies, darvadstrocel was associated with sustained clinical remission up to 52, 104, and 156 weeks [48,49,50]. Based on these results, darvadstrocel obtained regulatory approval across several regions, including Europe, for the treatment of PFCD [51].

However, the US Food and Drug Administration insisted on a further placebo-controlled trial to be conducted in order to confirm/validate the findings. ADMIRE-CD II was conducted, which was a phase III randomized controlled trial (RCT) of 568 patients with PFCD, allocating patients to either darvadstrocel or placebo. Much to the disappointment of many patients and clinicians, darvadstrocel did not meet its primary endpoint of superiority for combined remission at week 24 versus placebo (estimated treatment difference: 2.4%; 95% CI, −5.8 to 10.6; *p* = 0.571) [51]. Indeed, as well as the lack of benefit for the primary endpoint, there was also a lack of statistical difference against placebo for all of the secondary endpoints [52]. As a result of ADMIRE-CD-II, the sponsor requested global withdrawal of marketing authorization for darvadstrocel, meaning that to date, there are no currently licensed MSC therapies for IBD.

The discrepancy between the results of ADMIRE-CD and ADMIRE-CD II warrants further exploration [53]. Although there are multiple reasons, one likely important factor for the lack of benefit seen for MSCs in ADMIRE-CD-II was the high “placebo” response rate observed. It is important to note that the “placebo” control arm in ADMIRE-CD-II actually had multiple active interventions, including examination under anesthesia, curettage of internal fistula tract openings followed by preoperative seton placement, as well as >90% of patients receiving biologic therapy, often in combination treatment with immunosuppressants. In addition, the broad inclusion criteria applied in the ADMIRE-CD II resulted in the majority of included patients having only a single tract fistula. The combination of all the above factors likely contributed to much higher than anticipated remission rates in the control group at week 24, and the lack of additional benefit being observed with darvadstrocel [54].

### 2.2. Hematopoietic Stem Cell Therapy

HSCs reconstitute the immune system after transplantation and are commonly used as a curative cellular therapy for a variety of hematological disorders [55]. The key distinction is whether HSC transplantation (HSCT) is autologous or allogeneic. The idea of using HSCT in the treatment of non-hematological diseases was based mainly on initial case reports of patients who achieved long-term remission of their autoimmune disease after HSCT was performed for their hematological disease. Subsequently, there has been substantial interest in using HSCT for immune-mediated inflammatory diseases (IMIDs), including systemic lupus erythematosus (SLE) and systemic sclerosis (SSc) [56].

The role of autologous HSCT is to eliminate dysregulated immune cells and replace them with uncommitted stem cells, aiming to generate a more tolerogenic immune cell repertoire [57]. Autologous HSCT consists of two phases: mobilization and conditioning. During the mobilization phase, HSCs are mobilized from the bone marrow to peripheral blood, collected by apheresis, and then cryopreserved [58]. In order to mobilize stem cells, cyclophosphamide is typically administered, followed by granulocyte colony-stimulating factor [59,60]. Leukapheresis is then performed when the CD34^+^ count reaches the targeted number of cells/kg of body weight. The conditioning phase involves the administration of high-dose chemotherapy and/or rabbit antithymocyte globulin (rATG) to induce immune ablation and T-cell depletion, followed by subsequent reinfusion of previously collected HSCs. Notably, conditioning has been considered the major driver of toxicity with HSCT. Although there is no risk of graft-versus-host disease, autologous HSCT does still carry the risk of recurrence due to persistence of genetic predisposition to disease and the possibility for reinfusion of pre-existing, self-reactive T cells.

The first RCT to use autologous HSCT in IBD was the ASTIC (Autologous Stem Cell Transplantation International Crohn Disease) trial. ASTIC randomized 45 patients from six European countries and was designed to evaluate the effect of autologous unselected HSCT compared with conventional therapy in patients with refractory CD, with the primary endpoint being assessed after 1 year [59]. Patients underwent mobilization using cyclophosphamide and filgrastim and were then randomized to receive HSCT immediately after successful mobilization or after one year (delayed) [59].

ASTIC failed to reach its primary endpoint—a very high bar of complete clinical remission defined by CDAI <150, with no use of corticosteroids, biologics, or other immunosuppressants and no evidence of endoscopic or radiological activity. However, when examining the secondary outcomes, there did appear to be signals of benefit for autologous HSCT, with 61% of patients able to stop taking immunosuppressive drugs vs. 23% in the control arm (*p* = 0.01) and 26% more patients being free of disease activity on imaging (*p* = 0.054) and with CDAI <150 (*p* = 0.052). A further analysis of the pooled data from ASTIC, after the administration of HSC to the patients from the delayed arm, showed that complete endoscopic healing was observed in 19/38 (50%) patients [61]. Importantly, though, a large number of serious adverse events (SAEs) were observed.

Given the potential signal of benefit but bearing in mind the toxicity of the regimen used in ASTIC, a subsequent trial (ASTIClite) was performed with a less intense conditioning regimen [60]. In ASTIClite, 23 patients were randomized to the intervention group (*n* = 13, 57%) or to the control group (*n* = 10, 43%). The primary outcome was absence of endoscopic ulceration (SES-CD ulcer sub-score of 0) without surgery or death at week 48. In patients with available data, the absence of endoscopic ulceration without surgery or death was reported in 43% of patients in the intervention group (3/7), but in none from the control group (0/6). However, once again, the autologous HSCT was shown to have a high rate of adverse events. There were nine reported serious adverse reactions in six (46%) patients. These included renal failure due to proven thrombotic microangiopathy in three participants and two deaths. One death occurred in a patient receiving autologous HSCT due to pulmonary veno-occlusive disease, while the second death occurred again in the intervention arm after week 48 and was attributed to thrombotic microangiopathy-related renal failure. ASTIClite trial was stopped early due to these safety concerns, and the authors concluded that the regimen used was unsuitable for future use in either clinical trials or clinical practice due to the significant adverse events observed.

Even though both ASTIC and ASTIClite have suggested signals of potential benefit, the high rates of adverse events and the seriousness of these adverse events underscore the major safety concerns with HSCT. Notable safety concerns, even with the low-dose regimen used in ASTIClite, included the development of thrombotic microangiopathy leading to renal failure and pulmonary veno-occlusive disease. The current status quo is therefore to only consider autologous HSCT in highly selected patients with refractory CD, where there are no other treatment options and where the patients are willing to accept the benefit-risk profile of this intervention. It is widely accepted, though, that such intervention should only be conducted within experienced tertiary centers, with hematological support/expertise and with reduced-intensity conditioning regimens to optimize safety [62,63].

In contrast to autologous HSCT, allogeneic HSCT is often the critical treatment in many cases of VEO-IBD, where patients may have refractory disease due to monogenetic predisposition to the disease [55,57]. A single-center retrospective study from Philadelphia showed that of 25 children with monogenic IBD who underwent HSCT, 92% achieved sustained medication-free remission, and 60% were able to undergo re-anastomosis of their stoma [64].

### 2.3. Reinvigorated Interest in B-Cell Targeting

IBD has traditionally been considered a disorder of T-cell, myeloid, and innate immune dysfunction, with B-cells thought to only play a bystander role [65]. This view is now increasingly being challenged. Mucosal biopsies from patients with IBD have demonstrated expansion of dysregulated B-cell and plasma-cell populations [66]. In addition, the therapeutic effect of vedolizumab is thought to depend in part on blocking α4β7 integrin-mediated lymphocyte trafficking to the intestinal mucosa [67].

However, results from the first B-cell depletion trial using rituximab, an anti-CD20 monoclonal antibody, showed no benefit over placebo in an unselected population of patients with moderate-to-severe ulcerative colitis [68]. This may be the case given that B-cell-driven pathology may define only a subgroup of patients with IBD rather than the disease as a whole. Two lines of evidence have helped support this hypothesis. First was the identification of neutralizing autoantibodies against interleukin-10 (IL-10) in a child with infantile-onset IBD phenocopying inborn errors of IL-10 signaling. B-cell depletion, using rituximab, reduced the anti-IL-10 titer and permitted withdrawal of conventional therapy [69]. Extending this to an adult cohort, neutralizing anti-IL-10 autoantibodies were detected in approximately 3.5% of patients, and seropositivity was strongly associated with the HLA-DRB1*01:03 risk allele [70]. This allele was independently associated with earlier initiation of advanced therapy and higher risk of advanced-therapy failure across IBD phenotypes [71]. There have also been several new advances in B-cell depletion, with more targeted and effective options [72,73]. Together, these findings offer hope for future B-cell depleting strategies. A biomarker-defined subgroup such as anti-IL-10 autoantibody seropositivity, potentially enriched by HLA-DRB1*01:03 genotyping, might offer a rational basis for precision-targeted B-cell depletion. Although these anti-IL-10 autoantibody and HLA-DRB1*01:03 findings appear to have clinical relevance, the studies to date do not demonstrate that either of these biomarkers would predict response to B-cell depleting therapy. A biomarker-defined subgroup such as anti–IL-10 autoantibody seropositivity, potentially enriched by HLA-DRB1*01:03 genotyping, might offer a rational basis for precision-targeted B-cell depletion. This is a hypothesis that would warrant further exploration in future dedicated experimental medicine and biomarker-guided studies.

## 3. CAR-T Cells in IBD

**Figure 2:**
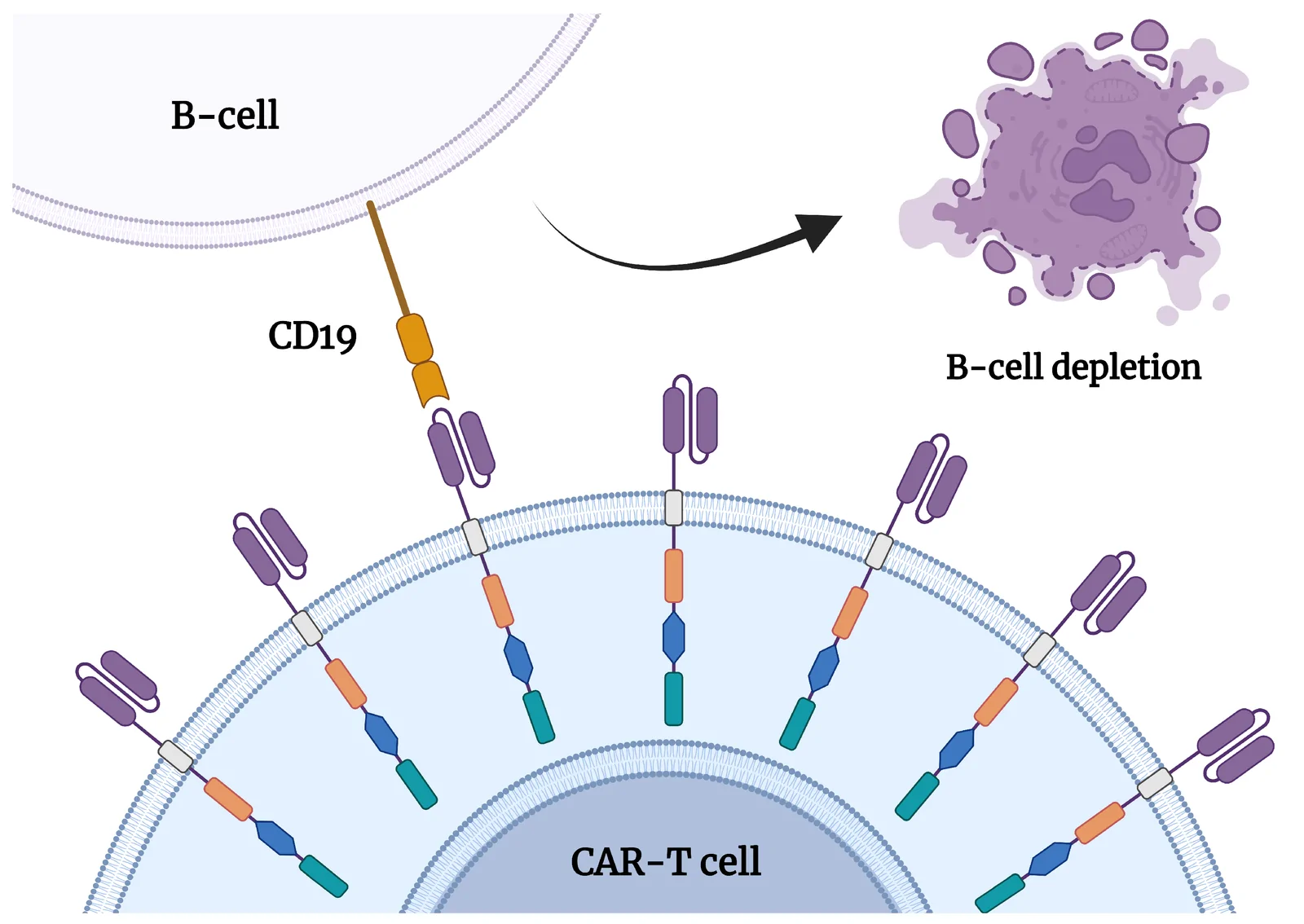
CAR-T cell therapy in IBD.

CARs are engineered proteins with antigen-binding and effector functions, which are introduced into T cells or natural killer (NK) cells, where they are expressed on the cell surface (Figure 2). CAR-expressing cells were initially developed for the treatment of hematological cancers and demonstrated high levels of success [74,75].

Given their success in hematology and oncology, CARs have been an intense area of research focus in recent years in IMIDs, particularly given their ability to selectively deplete B-cells and to reset the immune system [76,77]. However, there is a fundamental difference in the therapeutic objectives between their use in the treatment of cancer and IMIDs. In oncology and hematology, CAR-T cells act as cytotoxic effectors, whose goal is persistent immune surveillance. On the other hand, in IMIDs the role of CAR-T cells is to interrupt the pathogenic immune circuits, to “reset” the immune system and restore self-tolerance [78]. One notable advantage of using CARs in IMIDs would be that a single infusion of CAR-T cells may provide complete/long-lasting depletion of B-cells, which may not be possible to achieve with many immunosuppressive alternatives.

Initial application of CAR-T cell therapy across a range of IMIDs demonstrated highly promising short-term findings [79,80]. Then, in 2025, a case report was presented of a 21-year-old female with severe multidrug-resistant UC, successfully treated with a transfer of autologous CAR-T cells [81]. After the failure of ten different medications to control inflammation and the patient’s refusal of colectomy, the CD19-directed T-cell engager blinatumomab was administered in three doses. However, this treatment resulted in only a transient amelioration of disease activity and did not lead to a sustained depletion of B-cells.

Given an initial transient response, the treating team felt there was sufficient rationale to aim for deeper levels of B-cell depletion with CAR-T cell therapy. The patient was administered a standard lymphodepleting chemotherapy regimen, followed by an infusion of 1 × 106 autologous CD19 CAR-T cells per kilogram of body weight. The CAR-T cells rapidly expanded and resulted in peripheral and mucosal B-cell depletion, with clinical and biochemical remission. Notably, in follow-up, the patient had sustained remission without the need for any concomitant therapy and with resumption of normal activities of daily living. Deep healing was confirmed by endoscopy, histology, and ultrasound.

Although this case report provides evidence of clinical plausibility, coupled with the promising evidence observed from other analogous IMIDs, a single case report in IBD can not be considered as definitive proof of either efficacy or effectiveness. A single patient achieving drug-free remission and short-term endoscopic healing provides evidence of clinical plausibility, but not of long-term, durable efficacy and safety. In this regard, it is necessary to clearly state that CAR-T-cell therapy in IBD should still be considered only as having preliminary proof-of-concept, and is far from an established treatment to consider as part of routine standard-of-care clinical pathways.

It is also important to highlight that multiple potential adverse events have been reported with CAR-T cell therapies, including risks of serious infection, increased long-term risks of malignancy, as well as potentially life-threatening toxicity with cytokine release syndrome (CRS) and immune effector-cell-associated neurotoxicity syndrome (ICANS). Given the serious and potentially life-threatening nature of these adverse events, it is important for detailed risk–benefit discussions to take place with patients and for all of these factors to be considered before patients consent to administration of CAR-T-cell therapy. This is particularly pertinent in UC, where surgical intervention with a colectomy represents a relatively safe treatment option for patients, and one which has a much greater evidence base to support as a current standard of care option for patients with refractory disease. Reassuringly, in this reported case of refractory UC, only a mild (grade 1) cytokine release syndrome was reported with CAR-T therapy, and with spontaneous resolution. In addition, asymptomatic hypogammaglobulinemia and a transient episode of neutropenia were successfully treated with a single dose of granulocyte colony-stimulating factor.

The evidence discussed above regarding endotypes with neutralizing autoantibodies and the associated genetic haplotype of HLA-DRB*01:03 may have implications for clinical decision-making and help identify patients who might benefit most from CAR-T-directed treatments in the future.

An additional important advance specifically for CAR-T cells in IBD has been the recognition that it is possible to engineer CAR-T regulatory cells specifically targeting disease-relevant proteins such as the interleukin-23 receptor [82]. This has shown promise across in vitro assays and in a xenograft tumor model. The main advantage of these CAR-Tregs is that they can achieve an effect on target cells expressing the interleukin-23 receptor, without affecting the endogenous function of either interleukin-23 or its receptor.

## 4. Regulatory T Cells

**Figure 3:**
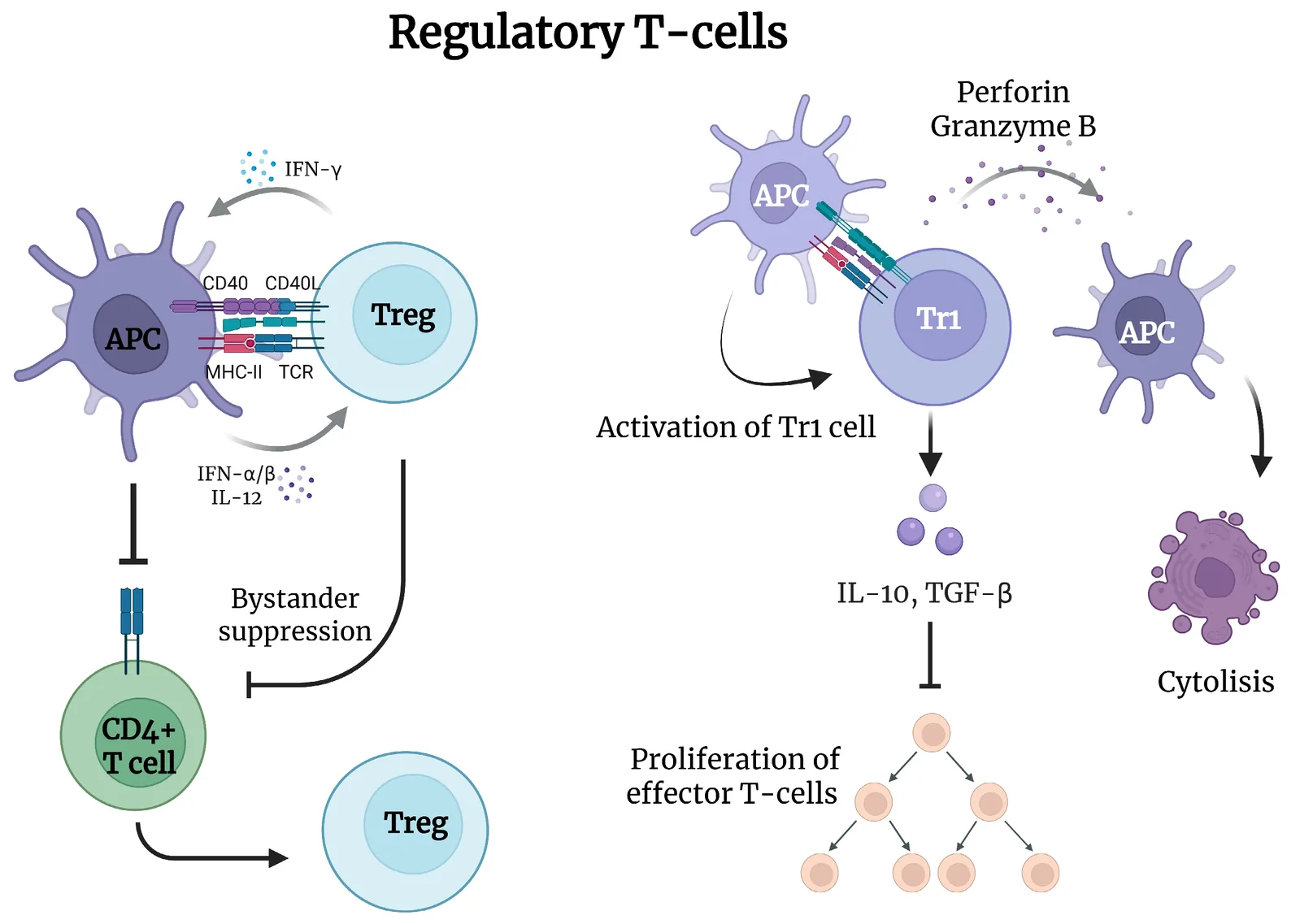
Regulatory T-cell therapy in IBD.

Regulatory T cells (Tregs) have also been directly targeted. Tregs are CD4+ T cells characterized by the expression of CD4, IL-2 receptor α chain (CD25), and the master transcription factor forkhead box P3 (Foxp3) [83]. Tregs are often categorized based on their origin into natural or thymic Tregs (nTregs or tTregs), peripherally induced Tregs (pTregs), and in vitro induced Tregs (iTregs) [84]. They are key mediators of peripheral immune tolerance, particularly in the intestine [82]. The role of Treg cells as guardians of the immune system can be attributed to their ability to restrain all currently recognized major types of inflammatory responses by modulating the activity of a wide range of cells of the innate and adaptive immune system [85]. The pool of regulatory T cells is quite heterogeneous and includes various cell types, including the two best-characterized subsets of CD4+FoxP3+ Tregs and Type I (Tr1) cells (Figure 3) [21].

IBD has long been recognized as a condition associated with Treg dysfunction, specifically with the loss of lineage stability, impaired suppressive function, and disrupted balance with pro-inflammatory T helper type 17 (Th17) cells [83,86]. Notably, levels of CD4+ CD25 high and FoxP3+ Tregs are increased during remission phases but diminish during active disease in patients with IBD [87,88].

The importance and efficacy of regulatory T cells in the suppression of inflammation in the gastrointestinal tract were first described in several animal models [89,90]. The first clinical trial based on the use of Tregs in patients with IBD investigated the effect of administration of autologous Tregs activated with ovalbumin (OVA) antigen. This trial was a 12-week, open-label, multicenter, single-injection, escalating-dose, phase 1/2a clinical trial, which included 20 patients with refractory CD [91]. All patients included had a previous inadequate response or intolerance to conventional treatments and anti-TNF agents. Overall, the treatment was well tolerated, although 55% of the patients experienced AEs considered to be related or possibly related to the study treatment, and seven patients experienced SAEs. In terms of efficacy, there was a clinically significant reduction in CDAI scores in 8/20 (40%) of patients at weeks 5 and 8. Notably, the effect was stronger in the group of patients who received the lowest dose of the Treg cells.

Subsequently, there has been major interest in expanding Tregs ex vivo and then administering them to patients to treat inflammation. Expansion ex vivo was demonstrated to be both feasible and scalable [92], and alongside work showing Treg defects present in patients with refractory CD [93], a proof-of-principle phase 1/2a clinical trial is now underway (TRIBUTE) to assess the feasibility and safety of administering autologous ex vivo expanded Tregs to patients with refractory CD (NCT03185000) [94].

Tr1 cells are another distinct group of regulatory T cells, characterized by high production of IL-10 and TGF-β1 but lack of FoxP3 expression [95,96]. Although Tregs and Tr1 share some similarities, there are key differences between these cells based on their origin and transcription factors driving development, secreted cytokine profiles, and their subsequent mechanism of immune modulation. Tregs are also highly stable under steady-state conditions, whereas Tr1 cells have more context-dependent stability, with reliance often on continuous IL-10 exposure to maintain their regulatory activities. Tr1 cells do not develop in the thymus as natural Tregs but differentiate in the periphery from naive T cells upon antigen exposure and under tolerogenic conditions driven by IL-10 or IL-27 [97]. A reduction in IL-10 production by mucosal Tr1-like cells has been observed in colonic biopsies from patients with both UC and CD, suggesting a potential benefit of exogenous administration of Tr1 cells [98]. Although previous subcutaneous administration of human recombinant IL-10 failed to show efficacy in patients with IBD, it has been postulated that this was likely due to a suboptimal route of delivery and short half-life [99,100], and that Tr1 cell administration may be a more effective method to increase IL-10 levels. Recently, an increase in IL-10 levels was shown to be feasible with Tr1 cell administration [91,101,102], and a dedicated phase 1/2a open-label trial (RESTORE) is currently ongoing to assess TRX103, an allogeneic TR1 cellular therapy for the treatment of refractory CD (NCT06721962).

## 5. Conclusions

In summary, cellular therapies are a potentially transformative group of interventions to treat IBD. Rather than simply suppressing the immune system, they offer a potentially paradigm-shifting mechanistic shift towards immune “reset” or “retuning”. There are multiple potential cellular therapies at various stages of investigation and evaluation in IBD, including autologous and allogeneic stem cells, CAR-T cells, and regulatory T cells (Supplementary Table S1).

Despite their promise, significant challenges and hurdles remain before cellular therapies can be routinely used in clinical practice. Robust, adequately powered, and high-quality clinical trials will be needed to determine efficacy and which populations might benefit the most from these interventions. Safety concerns, well-illustrated by high rates of treatment-related toxicity and adverse events in stem cell trials, mean that cellular therapies require rigorous protocol development, close monitoring and investigation in clinical trials, and should likely take place only in specialist centers with experience and expertise in administering cellular therapies. Moreover, variable efficacy, poor cell engraftment, complex manufacturing processes, and high financial costs present ongoing challenges for healthcare systems. Moving forward, optimizing patient selection through genetic and/or immunological profiling, and refining cell-engineering strategies, will be essential to translate cellular therapies into safe, sustainable, and accessible treatments in IBD.

## Data Availability

No new data were created or analyzed in this study. Data sharing is not applicable to this article.

## Supplementary Materials

The following supporting information can be downloaded at: https://www.mdpi.com/article/10.3390/jpm16100517/s1. Supplementary Table S1: Cellular therapies covered in this article.

## Abbreviations

The following abbreviations are used in this manuscript:

Anti-TNF	Antibodies to tumor necrosis factor
CAR	Chimeric antigen receptor
CD	Crohn’s disease
CDAI	Crohn’s disease activity index
CRS	Cytokine release syndrome
EVs	Extracellular vesicles
HLA	Human leukocyte antigen
HSC	Hematopoietic stem cell
IBD	Inflammatory Bowel Diseases
ICANS	Immune effector-cell–associated neurotoxicity syndrome
IPAA	Ileal-pouch anal anastomosis
MSC	Mesenchymal stem cell
PFCD	Perianal fistulizing Crohn’s disease
RCT	Randomized controlled trial
SAE	Serious adverse event
SLE	Systemic lupus erythematosus
SSc	Systemic sclerosis
UC	Ulcerative colitis
VEO-IBD	Very early onset IBD
