# Supplementary material for: Cellular Therapy in Inflammatory Bowel Diseases
Source: J Pers Med. 2026 Oct 3;16(10):517. doi: 10.3390/jpm16100517 (PMC13705663; doi:10.3390/jpm16100517)
Supplement: Supplementary file 1 [file jpm-16-00517-s001.zip › jpm-4547162-supplementary.pdf]

Supplementary Table S1: Cellular therapies covered in this article.

	**Mesenchymal stem cell therapy**		**Autologous haematopoietic stem cell therapy**	**Allogenic haematopoietic stem cell therapy**	**CD19 CAR-T cells**	**Conventional Tregs / CAR-Tregs**	**Tr1 cells**
**Origin**	Autologous / allogeneic		Autologous	Allogeneic	Autologous	Autologous	Autologous / allogeneic
**Route of administration**	Systemic i.v.	Local injection	Systemic i.v.	Systemic i.v.	Systemic i.v.	Systemic i.v.	Systemic i.v.
**Mechanism of action**	Secretion of bioactive molecules, cell-to-cell interactions paracrine signaling, induction of secondary immunological tolerance, signaling via apoptotic pathways		Elimination of dysregulated immune cells, replacement of them with uncommitted stem cells, with the aim of generating a more tolerogenic immune cell repertoire	Elimination of genetic risk	B-cell depletion, pathogenic immune circuits interruption, immune system "reset", restoration of the self-tolerance	Modulation of activity of immune cells, mediation of immune tolerance, suppression of inflammatory response	Cytokine secretion, IL-10 and TGF-β production, inhibition of proliferation of effector T-cells
**Target IBD population / phenotype**	UC / luminal CD	PFCD	luminal CD	monogenic IBD, VEO IBD	UC	luminal CD	luminal CD
**Principal safety concerns**	Serious AEs uncommon	Usually only mild AE - perianal sepsis, postoperative pain	Toxicity from chemotherapy, immune ablation and T-cell depletion; risk of recurrence due to persistence of genetic predisposition to disease, possibility for reinfusion of pre-existing, self-reactive T-cells, development of thrombotic	GVHD, infection, veno-occlusive disease	Serious infection, increased long-term risks of malignancy, potentially life-threatening toxicity with cytokine release syndrome (CRS) and immune effector-cell–associated neurotoxicity syndrome (ICANS)	Generally well tolerated, CD flare	Generally well tolerated

			microangiopathy leading to renal failure and pulmonary veno-occlusive disease				
**Highest level of clinical evidence**	Phase I/II trials	ADMIRE-CD, ADMIRE-CD II (randomized, double-blinded, placebo-controlled phase III trials) + extension trials	ASTIC, ASTIClite (phase III RCTs)	Retrospective trials	Single case report	Open-label, multicenter, phase 1/2a clinical trial	Murine models, ongoing phase 1/2a open-label trial
**Regulatory / developmental status**	Experimental	Approved by EMA 03/2018, withdrawn 12/2024	Stopped due to safety concerns	Used in highly-selected patients	Experimental	Experimental	Experimental
